# Supplementary material for: Are carbon emissions trading and green financial instruments synergistic? -Comprehensive quantitative research based on content analysis
Source: PLoS One. 2024 Mar 7;19(3):e0298601. doi: 10.1371/journal.pone.0298601 (PMC10919601; doi:10.1371/journal.pone.0298601)
Supplement: S1 Data — (ZIP) [file pone.0298601.s001.zip › Data/地级市Z_F工作报告.zip!/─┌├╔╣┼/░═╤σ─╫╢√╩╨/└┤╫╘╣1⁄2╓┌║┼í╛┬φ┐╦╩2╛▌═°í┐_2015.docx]

各位代表：

　　现在，我代表市人民政府向大会报告工作，请予审议，并请市政协委员和列席会议的同志提出意见。

　　一、2014年工作回顾

　　2014年是巴彦淖尔发展进程中具有重要里程碑意义的一年。在市委的坚强领导下，我们深入学习贯彻习近平总书记系列重要讲话和考察内蒙古重要讲话精神，全面落实自治区“8337”发展思路，大力弘扬总干精神，守望相助，团结奋斗，在赶超崛起的道路上实现了精彩跨越。

　　——绿色发展理念正在深入人心。绿色有机农牧业、清洁能源、黄河旅游等产业跃上新台阶，一批打基础、利长远的重大项目建成投产，被列为全国首批生态文明先行示范区，低碳、健康、文明的生活方式蔚然成风。

　　——“水、绿、文化”彰显独特魅力。中心城区和县城面貌焕然一新，美丽乡村建设走在全区前列，生态宜居、干净整洁、秩序良好的城市形象正在形成，草原水城、塞上绿城、河套文化名城展开秀美画卷。

　　——发展活力和动力不断增强。简政放权、政府职能转变取得重要成果，各级政府工作效率不断提高，群众办事更加方便快捷，社会投资和创业热情竞相迸发，地区发展环境明显改善，为新一轮发展奠定了基础、增添了后劲。

　　——幸福和谐的局面进一步巩固。15件民生实事全部完成，基本公共服务体系不断完善，一大批关系群众切身利益的热点难点问题得到解决，人民群众幸福感显著增强，民族团结、边疆安宁的风景线更加亮丽。

　　——对外知名度和影响力大幅提升。高水平接受了自治区推动科学发展观摩检查，成功承办了23个自治区现场会，70多批次考察团前来观摩学习，各项工作受到了自治区党委、政府和各盟市、厅局的高度评价，赢得了广大群众的普遍赞誉。

　　这些成绩，充分展现出巴彦淖尔的美好前景，极大增强了全市各族干部群众的自信心和自豪感，必将激励我们在新的征程上奋勇前进。

　　一年来，我们主要做了以下工作：

　　一是积极应对下行压力，经济发展稳中向好。把稳增长放在突出位置，着力抓项目、扩投资、扶企业，促进经济企稳回升。预计生产总值完成902亿元，增长8%；固定资产投资完成931.7亿元，增长16%；公共财政预算收入完成62亿元，增长7%；社会消费品零售总额完成212.4亿元，增长11.5%。粮食总产量达到66.7亿斤，新增设施农业4.59万亩，总面积达到20.5万亩。牧业年度羊饲养量首次突破2000万只，其中出栏1090万只，居全区第一。成功承办全国首届肉羊产业大会，被中国畜牧业协会授予“中国肉羊（巴美）之乡”称号。有机奶产量达到17.5万吨，占全国一半以上。工业经济在困境中平稳增长，预计规模以上工业增加值增长10.5%。包钢甲醇、盾安多晶硅二期、蒙羊肉羊精深加工等项目建成投产，黑猫煤化工、宇乐化工、圣牧高科有机奶等项目加快推进。新增风电装机55万千瓦，累计达到225万千瓦，新增光伏发电30万千瓦，累计达到65万千瓦，风光电占电力装机的近60%，能源生产结构实现重大转变。市中小企业服务中心挂牌成立，3个小微企业创业园建成投用。环境保护力度加大，隐患排查落实到位，节能减排完成年度目标。河套湿地成为国家4A级旅游景区，入选全国优选旅游项目名录，单日接待游客最高达15万人次。万泉湖、纳林湖、走西口•万丰民俗村等一批特色旅游景区建成，中蒙跨境旅游线路开通。全年接待游客和旅游收入增幅分别达到18.7%、24.5%。临河保税物流园区公路港建成，羊畜产品电子交易中心交易额突破3000万元，全市首个特色农产品葵花籽在渤海交易所上市交易，番茄、籽仁产业被列为国家级外贸转型示范基地，农畜产品出口额达到3亿美元，居自治区首位。社会融资规模达到140亿元，累计15户企业（合作社）进入资本市场融资，实现了历史性突破。

　　二是全面深化各项改革，对外开放日益扩大。抓住制约经济社会发展的突出问题集中进行攻坚，全面深化改革实现良好开局。习近平总书记视察内蒙古时提出的三项重点改革有序推进，龙头企业与农牧民利益紧密联结机制的实施方案已经出台。行政审批制度改革取得重大突破，市级行政审批事项大幅减少，办理程序和审批时限总体压缩50%以上。商事制度改革成效凸显，新注册企业4000多户，增长2.9倍；新注册资本188亿元，增长4倍，创历史新高。“营改增”试点范围扩大，取消行政事业性、经营性收费18项，减轻企业负担近亿元。集体建设用地、宅基地使用权确权登记颁证工作全面推进，土地草牧场确权登记颁证试点工作有序开展。在全区率先组建市级公共资源交易平台，交易额突破100亿元。农垦管理体制改革平稳推进，食品药品和工商行政管理、卫生和人口计生、质监、人防、金融等部门的机构改革顺利实施。教育管理体制进一步理顺，临河区职业教育、特殊教育划归市级管理，市直6所中小学和幼儿园移交临河区属地管理。公交体制改革全面启动，临河城区70岁以上老年人免费乘坐公交车。农村综合改革、扩权强县、县级公立医院改革试点工作取得阶段性成效，生态文明、文化体制、社会管理等方面改革扎实推进。对外开放水平进一步提升，进出口总额达到26亿美元，增长82.5%。引进国内到位资金330亿元，实际利用外资超过1亿美元。向北开放桥头堡建设迈出重要步伐，甘其毛都口岸进口煤炭突破1150万吨，铜精粉达到79万吨，出口电力10亿度。

　　三是扎实推进城乡建设，基础设施不断完善。城建投资完成192.7亿元，实施了一批道路、供水、管网等工程，新建改造道路62.5公里，新增集中供热面积280万平方米、各类管网148.5公里、天然气入户1.8万户，改造老旧小区35个，集中整治城镇出入口14个，黄河水厂一期工程建成投运。争取到国家开发银行棚户区改造贷款27.27亿元。新增园林绿化面积9100亩，我市被评为自治区园林城市，前旗成为自治区园林县城。城市管理水平显著提高，在全区12个盟市城市建设管理年活动考核中获得第4名，实现了历史性跨越。加快推进双河新区建设，先锋新桥、万佳城回迁房等项目全面开工。实施白彦花、干召庙等19个重点集镇改造，11个镇入选全国重点镇，23个镇入选自治区重点镇。行政村规划编制实现全覆盖。投资23亿元，完成270个嘎查村“十个全覆盖”工程，高标准建设了临河奋斗、前旗阿日齐、中旗格日楚鲁、后旗蒙汉、杭后南园、五原刘四拉、磴口协成等一批示范点。完成林业生态建设63.5万亩，乌拉特草原生态持续改善，乌兰布和沙草产业基地新增8万亩，乌梁素海生态补水2亿立方米。130万亩高标准基本农田、50万亩节水改造项目加快实施，灌区排水改造完成沟道清淤整治3846公里。海流图至五原一级公路竣工通车，临哈高速、中旗通用机场开工建设。实施三盛公220千伏等15项输变电工程，电网架构得到质的提升。

　　四是持续改善人民生活，社会事业蓬勃发展。在财政收支矛盾突出的情况下，民生支出达145.5亿元，占公共财政预算支出的75.1%，增长了5个百分点。预计城镇居民人均可支配收入达到22140元，增长9%；农村牧区居民人均可支配收入达到13650元，增长13%。新增城镇就业1.4万人，农牧民转移就业16.5万人，城镇“零就业”家庭实现动态清零。扶持4323人成功创业，带动就业1.6万人，市创业就业孵化中心建成投用。城乡养老、医疗等保障标准稳步提高，农垦系统4601名困难群众纳入城乡低保，为26.9万低收入农牧户发放煤补1.6亿元。企业退休人员养老金月人均增加173.4元，2.4万名80周岁以上老年人享受高龄津贴。市综合老年养护院、老年公寓开工建设，14个日间照料中心、15处农村牧区互助幸福院、国家二级殡仪馆建成投用。扎实推进“三到村三到户”精准扶贫、“金融扶贫富民”等工程，3.3万贫困人口稳定脱贫。开工各类保障性住房12062套，签订棚户区改造征收协议5839户。实施农村牧区危房改造20627户，两个边境旗3869户牧民危房全部改造完成。新建、改扩建中心集镇公办幼儿园10所，市直民族教育园区一期工程基本完工。选派240名优秀教师、大学生到农村牧区支教任教，为1076名新入学城乡低保家庭大学生发放资助金3742万元。河套学院新校区、新区市医院完成主体工程。成功举办全国沿黄河公路自行车赛、河套文化艺术节、河套湿地龙舟赛、环镜湖市民马拉松赛等文体活动，深受广大群众欢迎和好评。对外文化交流不断深入，开展了中蒙俄“同根同源”阴山岩刻艺术作品展，我市被命名为“中国观赏石之城”。建成全区首个农牧业科技信息移动服务平台、７个自治区级农牧业科技园区，辐射带动10万农牧民。9个“护城河”检查站建成投用，视频监控体系实现全覆盖，刑事案件、治安案件大幅下降，人民群众安全感进一步提升。安全生产、食品药品监管和质量监督不断强化。信访秩序持续好转，信访总量明显减少。民族宗教、双拥共建工作取得新进展，祖国北疆安全稳定屏障更加牢固。

　　五是加强政府自身建设，行政效能显著提升。认真落实改进作风各项规定，深入开展党的群众路线教育实践活动，“四风”问题得到有力整治，机关作风明显好转，干部精神面貌大为改观，社会风气为之一新，干部群众的向心力和凝聚力不断增强，形成了推进改革发展的强大正能量。扎实开展正风肃纪专项行动，全面清理和处置违规公务用车、超标办公用房、“吃空饷”人员，作风建设长效机制和刚性约束初步形成。坚决落实国务院“约法三章”，机构编制严格执行“红线”管理，全市“三公”经费压缩6.7%。接受了自治区党委巡视组的监督检查，反馈意见整改工作基本完成。认真执行市人大及其常委会决定，自觉接受市政协民主监督，政府班子主动上门征求人大、政协的意见和建议。办理人大代表建议91件，政协委员提案283件，办复率达100%，满意率达98%。“六五”普法深入开展，政府法制工作进一步加强。审计、监察工作力度加大，对违纪违法案件进行了严肃查处，起到了明显的警示教育作用，反腐倡廉建设不断深入。

　　各位代表，事非经过不知难。过去一年，我们共同经历了前所未有的考验，走过了一段十分难忘的历程。面对诸多困难和挑战，全市上下埋头苦干，奋力拼搏，挥洒了辛勤的汗水，谱写了亮丽的篇章，巴彦淖尔站在了新的起点上。这些成绩的取得，是市委统揽全局、科学决策的结果，是市人大、政协加强监督、鼎力支持的结果，是全市各族干部群众齐心协力、不懈奋斗的结果。在此，我代表市人民政府，向所有关心支持巴彦淖尔建设与发展的同志们、朋友们，表示崇高的敬意和衷心的感谢!

　　成绩弥足珍贵，困难和问题也不容忽视。主要是：受经济下行等因素影响，我们不得不对“十二五”规划指标进行了调整；经济总量偏小、产业层次不高、带动力强的大项目缺乏，仍然是经济发展面临的主要矛盾；新的经济增长点培育不足，优势特色农畜产品还没有真正走出去，部分企业运营艰难，财政增收十分困难；民生领域还存在一些薄弱环节，上学、看病、养老、就业、出行、社区服务、物业管理、征拆回迁等方面还有许多群众不满意的地方；一些公职人员能力、素质不能适应新形势新任务的要求，不担当、不作为、慵懒散等现象仍然存在，“四风”问题还没有彻底根治，等等。问题所在就是工作方向。我们要紧盯不放，常抓不懈，久久为功，争取使这些问题得到有效解决。

　　二、2015年工作总体部署

　　今年是全面深化改革的关键之年，是全面推进依法治市的开局之年，也是全面完成“十二五”规划的收官之年。当前，我国经济正处于增长速度换挡期、结构调整阵痛期、前期刺激政策消化期“三期叠加”阶段，各种矛盾和问题相互交织，但总体向好的基本面没有改变，仍处于大有可为的重要战略机遇期。中央坚持深化改革和依法治国双轮驱动，为全面建成小康社会提供了强大动力和坚强保障。国家高度重视民族地区、“三农三牧”和生态环保产业发展，把内蒙古纳入了“丝绸之路经济带”建设范围，加之迎接自治区成立70周年和我市“五城同创”，将实施一批重大项目，这都为我们带来了新的发展机遇。我市综合竞争优势日益显现，各项事业有活力、有亮点、有跨越，干部群众有思路、有干劲、有创新。只要我们坚定信心，抢抓机遇，就一定能把来之不易的良好态势保持下去，把“赏心悦目”的美景在更宽更广的领域生动展现。

　　今年政府工作的总体要求是：认真贯彻党的十八届三中、四中全会、中央经济工作会议、自治区党委九届十三次全委会议和市委三届十次全委（扩大）会议精神，全面落实自治区“8337”发展思路，坚持稳中求进工作总基调，主动适应经济发展新常态，牢固树立持续发展、转型发展、协调发展、和谐发展理念，以“迎庆创城”为总抓手，大力弘扬总干精神，推动经济扩量提质，狠抓改革攻坚，加强依法治市，突出创新驱动，努力保障民生，促进经济平稳健康发展和社会和谐稳定。

　　主要预期目标是：地区生产总值增长8%左右，固定资产投资增长13%，社会消费品零售总额增长11%，公共财政预算收入增长6%，城乡居民人均可支配收入分别增长10%和11%以上。居民消费价格涨幅控制在3%左右。节能减排完成自治区下达的目标任务。

　　根据总体要求和预期目标，做好当前和今后一个时期的工作，要做到“五个准确把握”。一是准确把握经济发展新常态。正确认识新常态、主动适应新常态、积极引领新常态是做好各项工作的基本逻辑。我们一定要保持积极的态度和善谋勇为的劲头，敏锐捕捉、充分发掘新常态下蕴藏的新机遇，按照“市场要活、创新要实、政策要宽”的要求，不断发现和培育新的经济增长点。要科学把握速度变化、结构优化、动力转换的新特点，保持经济在合理区间运行。今年，我市的主要预期目标达到或高于自治区水平，是积极适应新常态、权衡各种因素后确定的，体现了稳中求进的总基调，兼顾了增长换挡和产业升级的双重要求，需要全市上下经过艰苦努力才能实现。二是准确把握改革创新新要求。深化改革是适应经济发展新常态的关键之举。当前改革已进入攻坚期和深水区，必须拿出更大的勇气和力度，全面深化各领域改革，放开市场这只“看不见的手”，用好政府这只“看得见的手”，让全社会创造潜力充分释放。三是准确把握法治建设新任务。法治是安邦固本的基石。要全面推进依法治市，坚持法律面前人人平等，加快建设职能科学、权责法定、执法严明、公开公正、廉洁高效、守法诚信的法治政府，积极营造办事依法、遇事找法、解决问题靠法的社会氛围，推进各项工作法治化。四是准确把握民生改善新内涵。经济发展进入新常态，民生工作体现稳步、精准、公平等特点。要坚持“守住底线、突出重点、完善制度、引导舆论”的基本思路，更加注重保障基本民生，更加关注低收入群众生活，更加重视维护社会大局稳定，让改革发展成果更多更公平地惠及全市人民。五是准确把握生态文明新理念。良好的生态环境是巴彦淖尔最大的优势。要全面落实主体功能区规划，套区发展农畜产品加工、高新技术等绿色产业，沿山发展冶金、化工、电力等产业。要毫不放松地抓好生态保护和建设，最大限度增加生态资产、减少环境负债，推动形成绿色低碳循环发展新方式。

　　三、2015年重点工作

　　（一）推动重要领域改革取得突破。深化经济体制改革。完成五原县土地承包经营权确权登记颁证试点工作，其他旗县区各确定3个村组开展试点。坚持依法、自愿、有偿原则，引导土地草牧场经营权有序流转，健全公开规范的土地草牧场流转市场，发展适度规模经营。积极探索农村牧区宅基地、温室、棚圈等资产抵押融资。创新龙头企业和农牧民利益联结机制，因地制宜推广多种利益联结模式，让农牧民从产业化经营中得到更多实惠。稳步推进河套灌区水利体制改革，抓好农业水价综合改革。加快实施财税体制改革，认真执行《预算法》，严控预算增加，所有预算单位预决算和“三公”经费全部公开。强化政府性债务管理，严控新增债务。力争“营改增”实现全覆盖。深化金融体制改革，完成中旗、后旗、磴口农村信用联社股份制改造。实施不动产登记制度改革。做好杭锦后旗自治区级扩权强县试点工作，适时启动市级扩权强县试点。探索创新对蒙长期交流合作机制，尽快在我市设立蒙古国领事办事处，扩大商贸往来和人文交流。抓紧开展编制自然资源资产负债表、自然资源离任审计试点工作，探索建立生态环境损害责任终身追究制度，推进生态文明建设制度化、法治化。推进行政体制改革。继续简政放权，坚持放管结合，进一步激发市场活力。建立行政权力清单制度，6月份完成市本级行政权力梳理审核，力争年内在全市推开，实现“行政权力进清单、清单之外无权力”。大力推进文化广电、市场监管、医疗卫生等方面综合执法，全面落实行政执法责任制，加强执法监督。完成旗县区政府机构改革，深化事业单位改革。推进农垦后续改革，妥善解决遗留问题。统筹抓好其他领域改革。扎实推进教育体制改革，扩大优质资源覆盖面。加快县级公立医院改革步伐，健全就业创业和社会保障机制。推动国有经营性文化单位转企改制，完善新闻媒体管理和运行机制，做好新媒体与传统媒体融合发展试点工作。健全互联网管理体制机制。完善社会征信体系。推进户籍制度、公交体制改革。加快社会治理体制创新，深化涉法涉诉信访制度改革。支持司法体制机制改革，促进公正司法。

　　（二）促进工业经济扩量提质。强化工业主导地位不动摇，坚持扩总量、调结构、转方式并举，注重发挥信息化的引领作用，提高工业经济质量效益。规模以上工业增加值增长10.5%。全年工业完成投资360亿元，实施重点项目172项。开工建设祥光20万吨铜冶炼、神华一期填平补齐、东立光伏太阳能电池组件等项目，加快实施黑猫煤化工、宇乐化工及自备电厂、天昱园二期、华英世通云计算等项目，河套酒业技改、圣牧高科扩建、华拓矿业铁矿石采选、力诺太阳能发电等项目建成投产。积极推进中鑫400万吨煤制油、盾安三期及自备电厂、神东低热值煤发电、富量矿业钼矿采选、众兴油页岩采选等项目前期工作，力争早日落地。加快百万千瓦风电基地建设，风光电指标与重大项目捆绑实施、科学配置，提高资源利用率。认真落实扶持企业发展的政策措施，抓好工业经济运行调度，及时帮助企业解决困难和问题。加快推进工业与信息化融合，引导企业加大科技创新和技改力度，推动传统产业改造升级，大力发展非资源型产业。完善园区供电、绿化、排污等基础设施，打造绿色工业园区。加强中小企业创业园建设，扶持小微企业发展，培育壮大企业家队伍。牢固树立“招商决定项目、项目决定税收、税收决定财力”的理念，切实加大招商引资力度，按照国家产业政策导向，严把引进项目关，有选有弃，引进市场前景好、技术含量高、发展潜力大的项目，增强可持续发展后劲。推动甘其毛都口岸基础设施互联互通，做好跨境经济合作区前期工作，加快电子口岸、铜精粉出入境专用通道建设，启用互市贸易区，建成重载公路，过煤量突破1300万吨、铜精粉突破100万吨，争取更多的过境资源落地加工、转化增值。坚持源头严防、过程严管、后果严惩，加大环保违法行为查处力度，加强重点行业、重点企业节能降耗和污染治理，实现污水处理厂稳定运行、达标排放。

　　（三）大力发展绿色有机农牧业。坚持走产出高效、产品安全、资源节约、环境友好的现代农牧业发展道路，当好全区绿色农畜产品生产加工输出基地排头兵。农作物总播面积稳定在1000万亩以上，粮食产量保持总体稳定。鼓励引导合作社、龙头企业等经营主体在现有基础上集中连片发展设施农业，建设临河城关、五原八里桥、杭后头道桥等万亩示范基地，新增设施农业3.52万亩，同时不断提高利用率和收益率。加快实施新增出栏1000万只肉羊发展规划，建成5个年出栏10万只以上的肉羊养殖园区，肉羊饲养量达到2300万只。新建、改扩建规模化养殖场350个，新增有机奶牛1万头。大力推进肉羊产品精深加工，延长产业链，提升附加值。合理布局肉羊屠宰加工企业，严格控制新增产能，绝不搞盲目、低水平重复建设。引导企业加大原料基地建设，像爱护眼睛一样保护好河套特色品牌，重拳出击、严厉打击“挂羊头卖狗肉”行为。谁砸河套的牌子，就砸谁的“饭碗”，让造假售假者受到法律的严惩。充分发挥绿色无污染优势，积极申报乌拉特羊、河套小麦、磴口华莱士等国家地理标志产品登记保护。巩固和拓展河套农畜产品外销市场，抓好肉羊、葵花籽等电子交易平台建设，探索建立河套农产品交易中心，稳步扩大番茄、籽仁、脱水菜、羊绒等优质农畜产品出口，让好东西卖上好价钱。举办第二届全国肉羊产业大会，争取河套农畜产品进出口博览会、“新丝绸之路”农牧业科技成果博览会在我市召开。健全农牧业服务体系，抓好动物疫病防控和农畜产品质量安全可追溯体系建设，加强新品种、新技术试验示范推广，每个旗县区新建1—2个万亩高标准科技示范园区。加强种子工程建设，抓好葵花、玉米等农作物良种研发和繁育。培育新型职业农牧民，完成科技培训30万人次。实施100万亩高标准基本农田、50万亩节水配套改造、15万亩土地深耕深松等项目，完成河套灌区排水设施改造。支持山旱区发展节水农业，实施膜下滴灌25万亩。加快重点区域绿化，配套建设高标准农田防护林，新增经济林9万亩，育苗基地稳定在6万亩以上，完成林业生态建设70万亩。

　　（四）培育壮大服务业。新增规模以上服务业企业20家，服务业增加值增长6.6%。推进旅游与文化深度融合，打响河套旅游、草原旅游等特色品牌，推出乡村游、沙漠游、自驾游等旅游产品。启动乌不浪口抗日战争纪念园、磴口兵团文化博物馆、冯玉祥粮仓、黄河观凌塔建设，新建临河富强、前旗乌日图、五原农耕文化博览苑等民俗文化旅游项目。加快乌拉山国家森林公园、太阳庙沙漠、奈伦湖、乌拉特部落水上乐园等旅游项目建设，争取三盛公水利风景区建成4A级景区，推动万泉湖、纳林湖、公田村等景区提档升级。借助央视等高端平台，加大宣传促销力度，拓展客源市场，全年接待游客突破350万人次，实现旅游收入35亿元以上。加快发展金融业，引进各类金融机构2家以上，新增村镇银行、小额贷款公司、融资性担保公司5家以上。加强宏观政策引导，力争15家企业进入资本市场融资。大力发展商贸物流业，开工建设临河、前旗、后旗等工业园区铁路专用线，保税物流园区公路港实现通关运营。加快推进四季青菜市场东迁，启用萌业、八里桥农产品物流园，抓好临河城北客运站、陕坝客运站等项目。合理引导住房消费，促进房地产市场健康发展。适应个性化、多样化消费需求，大力发展信息、文化、健康养老、体育健身等新兴业态。制定电子商务发展规划，支持电商企业尽快做大，培育新的经济增长点。

　　（五）全力推进新型城镇化建设。本着尊重自然、因地制宜、顺势而为的原则，推动中心城区、县城、集镇、新农村新牧区协调发展，提高城镇化质量和水平。完善新型城镇化规划体系，严格规划管理。进一步做美中心城区，推进县城提质升级，加快双河新区建设步伐。加大城镇基础设施建设力度，打通影响百姓出行的“断头路”，新修街路30公里，完成城镇出入口改造9个，改造老旧小区80万平方米、背街巷道7万平方米。开工建设临河至包头天然气输送工程，加快经济开发区污水管网扩建，铺设各类管网127公里，新增集中供热面积180万平方米、天然气入户2万户。开工各类保障性住房18182套，用好用足国家开发银行棚户区改造贷款，推广居民“自主改造”新模式，完成棚户区改造1.38万户。坚持宜拆则拆、宜改则改，在条件较好的平房区，推行水、电、气、暖、路综合配套改造。专项推进临河龙湖湾和北边渠、杭后富霖华庭、前旗景香名苑、五原胜景苑等棚户区改造尾留工程，做好居民回迁安置工作。对棚户区已征收的土地和资产，摸清底数，登记造册，加强管理。推动城镇绿化向园艺化方向发展，全市新增园林绿地2300亩以上。强化城镇精细化管理，建设数字城市、“海绵城市”，开展县城地下市政管网普查建档工作。提高供热供水质量，规范物业管理秩序。稳步推进就地城镇化，充分尊重农牧民意愿，体现乡土特色和田园风光，积极发展庭院经济，加快建设美丽乡村。抓好大佘太、天吉泰等11个重点镇建设。实施480个嘎查村“十个全覆盖”工程，注重典型引路，捆绑项目资金，加大督查力度，力争完成全部任务量的90%以上，让这项重大民生工程更多更早地惠及广大农牧民。开展包兰铁路沿线绿化和环境综合整治，加大农村“四堆”清理力度，抓好11个垃圾污水处理示范村建设，建立健全村庄环境卫生长效管理机制。做好全市土地利用总体规划调整完善工作，清理闲置土地，盘活存量用地和资产。加快临哈高速、甘其毛都至临河一级公路建设，推进甘其毛都至海流图、青山至乌根高勒一级公路改扩建，新建、改建农村牧区公路1000公里。中旗通用机场建成投用。实施临河西220千伏等13项输变电工程。

　　（六）加强保障和改善民生工作。今年市和旗县区财政安排民生专项资金24.1亿元，增加1.1亿元。全力保持就业稳定，全年新增城镇就业1.25万人，农村牧区劳动力转移就业16万人。发放小额担保贷款2亿元，完成城乡职业技能培训2万人，支持河套学院、市职业技术学校等院校培育特色品牌。认真落实扶持创业就业的政策措施，安排1000万元创业就业发展基金，营造大众创业、万众创新的良好环境。新农合人均筹资标准提高到480元，城乡低保、五保供养等保障标准稳定在现有水平。多做雪中送炭的事情，让困难群众生活得到保障、心灵得到温暖。大力实施农村牧区低收入家庭1吨煤、城乡低保家庭新入学大学生每年资助1万元、城镇“零就业”家庭至少1人就业“三个一”工程。统筹用好5000万元专项资金，加大困难家庭大病救助力度，扩大救助范围，提高救助标准。建立社会救助申请综合性服务平台，让困难群众求助有门、受助及时。深入推进精准扶贫，认真落实领导干部联系贫困点制度、定点扶贫机制和“三到村三到户”等帮扶措施，重点抓好乌梁素海周边、乌兰布和沙区、边境牧区、黄河北岸、总排干沿线等贫困带扶贫工作，确保3万人稳定脱贫。对转移安置后生活困难的牧民，出台专项扶持政策，确保转得出、稳得住、能致富。

　　（七）统筹发展各项社会事业。实施第二期学前教育三年行动计划，新建、改扩建幼儿园18所。建设50所义务教育标准化学校，3个旗县通过义务教育均衡发展验收。启动市直民族教育园区二期工程，大力发展现代职业教育，河套学院新校区完工。加快新区市医院建设，完成市精神病院、5个苏木镇卫生院建设，实现“健康保障小药箱”进牧户全覆盖，提升基层医疗卫生信息化水平。扶持蒙中医药事业发展。推进心理健康服务体系建设，预防和减少心理问题。落实好计划生育“单独两孩”政策，稳定适度低生育水平。支持妇联、共青团、工会、残联等组织按照法律和章程开展工作，切实维护妇女儿童、职工、残疾人的合法权益。关注老年群体，加快老龄、慈善事业发展。弘扬中华传统美德，践行社会主义核心价值观。以迎接自治区成立70周年为契机，积极推进全国文明城、园林城、卫生城、双拥模范城、环保模范城“五城同创”。实施城乡公共文化服务惠民工程，办好全国沿黄河公路自行车赛、龙舟公开赛、摄影大赛等活动，创作《乌拉特西迁》等精品舞台剧，举办中蒙俄国际岩刻艺术作品展。加快实施“15分钟健身圈”规划，新建1处全民健身活动中心。大力发展足球运动，市财政安排1000万元专项经费，在市体校和各旗县区建设足球特色学校9所，办好各类足球赛事。促进科技成果转化和推广，力争创建1个国家级可持续发展实验区，新增2—3个自治区级企业研发中心和1家高新技术企业。充分发挥市农牧业科学院、河套农牧业技术研究院等科研机构作用，加快研发具有自主知识产权的核心技术。深入实施人才强市工程，统筹抓好各类人才队伍建设。深化平安创建，全面推广“三一四全”、“三联六调”工作法、村民代表常设制，完善社会治理机制，有效预防和化解社会矛盾。加强社会治安基础信息化、网络化建设，形成立体化打防控体系。扎实开展社区矫正和法律援助工作，预防和打击各类违法犯罪行为。规范社区居委会职能，完成第九届嘎查村委会换届选举。牢固树立安全发展理念，切实抓好生产安全、生态安全、食品药品安全、网络信息安全，加大隐患排查治理力度，加强应急管理，确保不发生严重暴力恐怖袭击、大规模群体性事件、重大安全事故和恶性网络炒作事件。支持国防和军队建设，全面落实党的民族政策，依法管理宗教事务，巩固民族团结、边疆安宁的大好局面。

　　（八）努力建设人民满意政府。政府的一切权力来自人民、源自法授。要坚持依宪行政、依法行政，严格按照法定权限和程序行使权力、履行职责，做到法定职责必须为、法无授权不可为。各级政府及工作人员要带头遵守宪法和法律，提高运用法治思维和法治方式推进工作的能力。健全依法决策机制，把公众参与、专家论证、风险评估、合法性审查、集体讨论决定作为重大行政决策的法定程序，建立重大决策终身责任追究制度及责任倒查机制。完善政府法律顾问和公职律师制度，加强法治队伍建设。自觉接受人大依法监督和政协民主监督，提高人大代表建议和政协委员提案办理质量，为人大、政协更好地开展工作提供保障。支持人民法院、人民检察院依法独立行使职权。广泛听取各民主党派、工商联、无党派人士和人民团体的意见建议，主动接受社会公众和新闻舆论监督。加强重大问题调查研究，科学编制“十三五”规划。巩固扩大群众路线教育实践活动成果，确保整改工作落到实处。深入开展“三严三实”专题教育，持之以恒落实改进作风各项规定，决不让“四风”反弹回潮、死灰复燃。大力倡导“马上就办”工作理念，坚决纠正慢作为、不作为、庸懒散等行为，严厉整治“门好进、脸好看，但事仍然难办”和变相吃拿卡要等歪风，推进机关效能建设常态化、长效化。积极推进干部素质提升工程，坚持理论培训和实践历练并举，加强履职尽责所需的现代经济、社会治理等知识的学习，不断完善知识结构，提高业务能力，增强向上争取、对外联络、服务群众的能力和本领，让各级领导干部真正成为新常态下做好本职工作的行家里手。全面推进政务公开，让权力在阳光下运行。完善四级便民服务体系，打造高效优质的政务服务平台。严格执行厉行节约、反对浪费的有关规定，严守国务院“约法三章”，从严控制“三公”经费，推进公务用车制度改革。强化审计监督和整改，逐步实现公共资金、国有资产、国有资源和领导干部履行经济责任情况审计全覆盖。严格落实“一岗双责”，强化党风廉政建设，严肃查处违纪违法案件，树立法治、高效、廉洁的政府形象。

　　各位代表，我们正处在一个有梦想、有机遇、有奋斗的新时代，一切美好的东西都能够创造出来。让我们更加紧密地团结在以习近平同志为总书记的党中央周围，在自治区党委、政府和市委的坚强领导下，大力弘扬总干精神，守望相助，团结奋斗，激情创业，砥砺前行，把巴彦淖尔打造成为祖国北疆亮丽风景线上的耀眼明珠！
